# Clinical Relationship Between Serum ApoB, HER2, and Myocardial Ischemia Risk in Breast Cancer Patients

**DOI:** 10.1002/cnr2.70075

**Published:** 2025-07-18

**Authors:** Yeyan Lei, Dongmei Li, Shuang Bai, Xing Zeng, Rongyuan Yang, Qing Liu

**Affiliations:** ^1^ Zhuhai Doumen Maternal and Child Health Care Hospital, Guangdong Provincial Hospital of Chinese Medicine‐Zhuhai Hospital, the Second Clinical School of Medicine Guangzhou University of Chinese Medicine Zhuhai China; ^2^ Department of Clinical Medicine The Third Clinical School of Guangzhou Medical University Guangzhou China

**Keywords:** ApoB and HER2, breast cancer, case–control study, clinical prediction model, myocardial ischemia risk

## Abstract

**Background:**

The risk factors and clinical prediction of cardiovascular comorbidities in patients with breast cancer have not been fully clarified.

**Aims:**

This retrospective case–control study was designed to investigate the factors affecting myocardial ischemia occurrence in breast cancer patients.

**Methods and Results:**

A total of 194 cases (144 breast cancer and 50 benign breast tumor patients) were included. Univariate and multivariable Cox regression found that ApoB, age, and HER2 were significant factors responsible for the myocardial ischemia occurrence in breast cancer patients. By comparing the significance of ApoB in breast cancer patients versus benign breast tumor patients, it was observed that ApoB and HER2 were crucial predictors of myocardial ischemia in breast cancer patients compared to those with benign breast tumors. These factors were utilized to construct the clinical prediction model, achieving a combined area under the curve (AUC) of 0.583. The decision curve analysis (DCA) indicated that the model‐predicted population, within a threshold ranging from 0.35 to 0.70, would experience a therapeutically clinical net benefit. Kaplan–Meier plot indicated that ApoB^high^ and HER2^+^ categories were high‐risk populations for myocardial ischemia in breast cancer patients, although there was no significant difference between ApoB^low^ and ApoB^high^ subgroups for the 3‐year disease‐free survival.

**Conclusion:**

We demonstrated that ApoB and HER2 were potential factors in predicting the myocardial ischemia occurrence in breast cancer patients. This study will help provide clinical evidence for the early prediction of cardiovascular comorbidities in breast cancer patients.

**Abbreviations**
Apo: apolipoprotein
AUC: area under the curve
BMI: body mass index
CVD: cardiovascular disease
DCA: decision curve analysis
DFS: disease‐free survival
ER: estrogen receptor
HDL: high‐density lipoprotein
HER: human epidermal growth factor receptor
KM: Kaplan–Meier
LDL‐C: low‐density lipoprotein cholesterol
Lp(a): lysophosphatidic acid
PCM: patents Chinese medicine
PR: progesterone receptor
RCS: restricted cubic spline
RCT: randomized controlled trial
ROC: receiver operating characteristic
TC: total cholesterol
TG: triglyceride

## 1 Introduction

Breast cancer is one of the most common malignancies affecting women globally, with a significant incidence and survival rate. Consequently, the number of breast cancer survivors has increased substantially [1]. Alongside the burden of breast cancer itself, there is growing concern about the increased susceptibility to cardiovascular disease (CVD) among these patients [2]. Various studies have reported a higher prevalence of CVD, CVD‐related mortality, and overall mortality among breast cancer survivors compared to those without breast cancer [3].

Multiple risk factors contribute to the development of CVD in breast cancer patients, including age, dyslipidemia, obesity, lower education levels [4, 5], hypertension [6], and a sedentary lifestyle [7].

Apolipoprotein B (ApoB) is a crucial component of triglyceride‐rich lipoproteins and low‐density lipoprotein (LDL). It facilitates the transport of cholesterol and triglycerides from the liver to peripheral tissues, including the heart and arteries [8]. Elevated levels of ApoB‐containing lipoproteins, particularly LDL, are strongly associated with an increased risk of atherosclerosis, potentially leading to heart disease and stroke [9]. Therefore, monitoring ApoB levels is essential in assessing cardiovascular risk and reducing cardiovascular morbidity and mortality. Human epidermal growth factor receptor 2 (HER2) amplification was found in about 20% of breast cancers. Currently, HER2 immunohistochemistry (IHC) is used for a screening test, and in situ hybridization is used as a confirmation test [10], with related criteria according to the American Society of Clinical Oncology (ASCO)/College of American Pathologists (CAP) guidelines. However, the precise mechanisms underlying this elevated risk remain incompletely understood. Alterations in serumology such as lipid indicators may play a crucial role in the development of CVD in breast cancer patients [11, 12, 13]. Nevertheless, the precise relationship between serumological indicator levels and the risk of myocardial ischemia in breast cancer patients, and whether lipid indicators are independent risk factors for myocardial ischemia risk prediction in this population, remains unclear.

To address these questions, we conducted this study and aimed to investigate the association between serum lipid levels and the risk of myocardial ischemia in breast cancer patients. Additionally, we explored the impact of clinical factors such as the clinical stage and pathological subtype of breast cancer on the incidence of CVD within this population. The results of this study will provide valuable clinical insights for the early prediction and management of cardiovascular comorbidities in breast cancer patients.

## 2 Methods

### 2.1 Ethical Approval

This retrospective case–control study was approved by the Ethics Committee of Guangdong Provincial Hospital of Traditional Chinese Medicine (No. ZE2023‐318).

### 2.2 Patient Source and Data Collection

The medical information of the enrolled patients was retrospectively collected at Guangdong Provincial Hospital of Traditional Chinese Medicine‐Zhuhai Hospital from 2014 to 2020. The medical record data were collected including demographic data (e.g., age, weight, height, BMI) and comorbidities (hypertension, hyperglycemia, hyperlipidemia) which presented basic characteristic and underlying diseases, breast cancer stages (clinical stages) and pathological categories (TNM categories, ER, PR, HER2, Ki67) which presented the breast cancer pathological conditions, and lipid‐lowering medication (Atorvastatin, Rosuvastatin, etc.) which presented the lipid levels. The expression level of HER2 protein was detected by IHC staining of breast cancer tissue. HER2 expression levels were usually classified into four levels 0, 1+, 2+, and 3+. These standards were typically proposed by the American Society of Clinical Oncology and the American Pathological Society. The hematological data of lipid levels (e.g., TC, TG, LDL, HDL, apoA1, ApoB, Lpa) were retrospectively collected at Guangdong Provincial Hospital of Traditional Chinese Medicine‐Zhuhai Hospital. These data were detected using methods such as optical colorimetry with a blood lipid analyzer in the laboratory department by a full‐time staff of the hospital.

The patients were divided into two groups based on the “low” and “high” expression levels of ApoB, both in breast cancer patients and benign breast tumor patients. The baseline level of ApoB was about 0.4–1.1 g/L, and we calculated the 95% upper reference limit [14] of the baseline level at about 1 g/L. Thus, the “low” and “high” ApoB levels were decided by comparing the ApoB levels with the threshold of the 95% upper reference limit.

### 2.3 Inclusion and Exclusion Criteria

Criteria for inclusion: Firstly, there should be a clear diagnosis for the patients, indicating whether they were breast cancer or benign breast tumors. Secondly, the information of demographic, clinical symptoms, comorbidities, breast cancer stages and pathological categories, lipid levels and lipid‐lowering medication, and electrocardiogram examination can be found in the medical records.

Criteria for exclusion: Firstly, patients with existing CVDs before inclusion were excluded. Secondly, cases lacking primary and secondary outcomes were excluded.

### 2.4 Primary and Secondary Outcomes Collection

The primary outcome was the occurrence of myocardial ischemia in breast cancer patients, the incidence and the duration of disease occurrence were collected for analysis. The secondary outcome was the 3‐year disease‐free survival of breast cancer.

### 2.5 Univariate and Multivariate Cox Regression Analysis

Regarding the two categories of clinical outcome, i.e. the occurrence of myocardial ischemia in breast cancer patients, binary logistic regression with univariate and multivariable analysis was conducted to screen the independent predictors, including demographic information, complications, and medications. The univariate model of binary logistic regression analysis was performed initially, and the statistically selected parameters and the parameters with well‐known clinical associations were used for multivariable model screening. The *p* < 0.05 in multivariate analysis were taken as potential independent predictors in the forest plot.

### 2.6 Construction of Clinical Prediction Model and Calibration

The nomogram of the clinical prediction model was constructed with the parameters of *p* < 0.05 screened out in the multivariable Cox regression. To assess the internal validity of the nomogram, the entire dataset was resampled using bootstrapping methods (*B* = 1000 iterations) to approximate sampling distributions. Subsequently, calibration curves were plotted to compare observed and predicted outcome probabilities. The ideal calibration curve possesses an intercept of 0 and a slope of 1, indicating perfect alignment. Furthermore, a receiver operating characteristic (ROC) curve was plotted to assess the predictive performance of the nomogram, and the area under the curve (AUC) along with sensitivity and specificity thresholds was computed to provide a comprehensive representation of the model performance. Finally, the decision curve analysis (DCA) of the nomogram was performed to evaluate the net clinical benefit of treatment efficacy based on bootstrap resampling.

### 2.7 Statistical Analysis

The medical data were collected from the hospital's electronic medical record system, and data were analyzed using SPSS (v26.0 Inc. Chicago, Illinois, USA) and R (v3.6.2, http://www.r‐project.org) software. Continuous data were expressed as mean ± standard deviation and tested for normal distribution using the Kolmogorov–Smirnov test. If the continuous data followed a normal distribution, a Student's *t*‐test for independent samples was used for comparison between the two groups. Otherwise, a Mann–Whitney *U* test was used. Categorical variables were represented by frequency and proportion (%) and intergroup comparisons were performed using the Chi‐square (χ^2^) test with or without continuity correction or Fisher's exact test. A *p* value of less than 0.05 was considered statistically significant. The forest plot, nomogram, ROC curve with AUC, RCS plot, calibration curve, DCA, and Kaplan–Meier (KM) plot were constructed by *R* software.

## 3 Results

### 3.1 Baseline Characteristics

**FIGURE 1:**
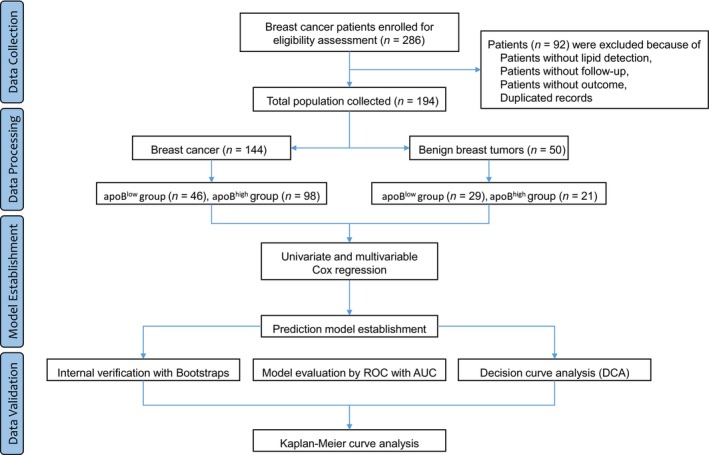
Flowchart for the data collection and analysis process.

According to the diagnosis of breast cancer, a total of 286 patients initially enrolled and 92 patients were excluded based on the criteria. Ultimately, 194 patients (*n* = 144 in breast cancer patients and *n* = 50 in benign breast tumor patients) were included in group allocation and the final analysis. The patients were divided into two groups based on the low and high expression levels of ApoB (*n* = 46 of ApoB^low^ and *n* = 98 of ApoB^high^ group in breast cancer patients, *n* = 29 of ApoB^low^ and *n* = 21 of ApoB^high^ group in benign breast tumor patients) (Figure 1 and Tables 1 and 2).

**TABLE 1:** Comparisons of the baseline characteristics in the groups with low or high levels of apoB in breast cancer patients, *n* (%) or median(IQR).

Variables	apoB^low^ group *N* = 46	apoB^high^ group *N* = 98	*p*
Demographics			
age	45.6 (10.1)	50.5 (10.6)	0.009*
weight	54.9 (7.64)	56.3 (7.29)	0.305
height	158 [155; 160]	156 [153; 160]	0.499
BMI	22.4 (2.86)	23.0 (2.76)	0.294
Comorbidities			
anemia	4 (8.70%)	3 (3.06%)	0.210
thyroid dysfunction	12 (26.1%)	35 (35.7%)	0.338
hypertension	3 (6.52%)	17 (17.3%)	0.135
hyperlipidemia	4 (8.70%)	23 (23.5%)	0.059
DM	1 (2.17%)	9 (9.18%)	0.169
No. of comorbidities			0.088
1	12 (26.1%)	31 (31.6%)	
2	3 (6.52%)	19 (19.4%)	
3	2 (4.35%)	6 (6.12%)	
TNM stages			
T			0.187
1	19 (44.2%)	39 (40.6%)	
2	10 (23.3%)	36 (37.5%)	
3	2 (4.65%)	6 (6.25%)	
4	2 (4.65%)	2 (2.08%)	
x	0 (0.00%)	3 (3.12%)	
*N*			0.069
1	8 (18.6%)	29 (30.2%)	
2	3 (6.98%)	9 (9.38%)	
3	4 (9.30%)	1 (1.04%)	
*M*			0.850
1	1 (2.33%)	2 (2.08%)	
*x*	1 (2.33%)	5 (5.21%)	
Clinical stage			
I	4 (9.30%)	8 (8.51%)	
IA	7 (16.3%)	18 (19.1%)	
IB	0 (0.00%)	2 (2.13%)	
IIA	10 (23.3%)	23 (24.5%)	
IIB	2 (4.65%)	21 (22.3%)	
IIIA	4 (9.30%)	7 (7.45%)	
IIIB	1 (2.33%)	1 (1.06%)	
IIIC	4 (9.30%)	1 (1.06%)	
IV	1 (2.33%)	3 (3.19%)	
Pathology			
ER+	0.60 [0.60; 0.67]	0.60 [0.60; 0.90]	0.188
PR+	0.30 [0.30; 0.30]	0.30 [0.10; 0.30]	0.713
HER2			0.702
1+	2 (4.35%)	9 (9.18%)	
2+	33 (71.7%)	68 (69.4%)	
3+	6 (13.0%)	9 (9.18%)	
Ki67+	0.70 [0.40; 0.70]	0.70 [0.20; 0.70]	0.403
Lipid			
TC	4.13 (0.81)	5.36 (0.81)	< 0.001*
TG	0.86 [0.66; 1.35]	1.48 [1.06; 1.89]	< 0.001*
HDL	1.52 [1.42; 1.67]	1.46 [1.17; 1.69]	0.201
LDL	2.47 (0.64)	3.46 (0.69)	< 0.001*
apoA1	1.56 [1.33; 1.77]	1.55 [1.46; 1.55]	0.514
apoB	0.76 [0.64; 0.87]	1.02 [0.98; 1.18]	< 0.001*
Lpa	125 [51.6; 232]	154 [93.5; 275]	0.051
Lipid‐lowering medication			
Atorvastatin	2 (4.35%)	19 (19.4%)	0.033*
Rosuvastatin	1 (2.17%)	8 (8.16%)	0.272
Simvastatin	3 (6.52%)	6 (6.12%)	1.000
Fluvastatin	0 (0.00%)	2 (2.04%)	1.000
Pivastatin	1 (2.17%)	2 (2.04%)	1.000
Ezetimibe	0 (0.00%)	1 (1.02%)	1.000
PCM	1 (2.17%)	2 (2.04%)	1.000
3‐year DFS	45 (97.83%)	95 (96.94%)	1.000
Myocardial ischemia	17 (37.0%)	50 (51.0%)	0.115
Arrhythmias	12 (26.1%)	37 (37.8%)	0.234

Abbreviations: BMI, body mass index; DFS, disease‐free survival; DM, diabetes mellitus; ER, estrogen; HDL, high‐density lipoprotein cholesterol; LDL, low‐density lipoprotein cholesterol; PCM, patent Chinese medicine; PR, progesterone; TC, total cholesterol; TG, triglyceride.

* *p* < 0.05.

**TABLE 2:** Comparisons of the baseline characteristics in the groups with low or high levels of apoB in benign breast tumor patients, *n* (%) or median(IQR).

Variables	apoB^low^ group *N* = 29	apoB^high^ group *N* = 21	*p*
Demographics			
age	37.8 (9.76)	40.6 (8.00)	0.270
weight	55.4 (6.69)	56.6 (5.85)	0.485
height	158 [158; 161]	158 [157; 160]	0.408
BMI	21.1 [20.2; 24.1]	22.6 [21.5; 24.3]	0.188
DM	0 (0.00%)	0 (0.00%)	—
Lipid			
TC	4.30 [3.86; 4.54]	5.04 [4.76; 5.50]	< 0.001*
TG	0.70 [0.52; 0.91]	0.97 [0.79; 1.27]	0.002*
HDL	1.61 (0.31)	1.55 (0.23)	0.482
LDL	2.41 (0.53)	3.71 (0.62)	< 0.001*
apoA1	1.53 (0.24)	1.53 (0.17)	0.993
apoB	0.73 (0.14)	1.14 (0.19)	< 0.001*
Lpa	184 [184; 184]	184 [184; 184]	0.972
Myocardial ischemia	4 (13.8%)	3 (14.3%)	1.000

Abbreviations: BMI, body mass index; DM, diabetes mellitus; HDL, high‐density lipoprotein cholesterol;LDL, low‐density lipoprotein cholesterol; TC, total cholesterol; TG, triglyceride.

* *p* < 0.05.

For the baseline characteristic analysis in the breast cancer patients, a significant difference was found in age (*p* = 0.009), lipids (TC, TG, LDL, ApoB) (*p* < 0.001), and Atorvastatin administration (*p* = 0.033). There were 21 patients who received atorvastatin (20 mg, qd, po) among the 144 breast cancer patients, with 2 (4.35%) in the ApoB^low^ group and 19 (19.4%) in the ApoB^high^ group (Table 1). For the other factors including BMI, comorbidities, TNM/Clinical/Pathological stages of breast cancer, there was no significant difference. Moreover, no significant difference was found in the 3‐year DFS. Although the statistical *p* value did not reach the criteria on the cardiovascular outcome of myocardial ischemia and arrhythmias, we found the occurrence of myocardial ischemia and arrhythmias were both increased in the ApoB^high^ group (Table 1). A similar baseline characteristic analysis was performed in the benign breast‐tuning patients (Table 2).

### 3.2 Screening the Factors Responsible for Myocardial Ischemia Risk in Breast Cancer Patients With Univariate and Multivariable Cox Regression

**FIGURE 2:**
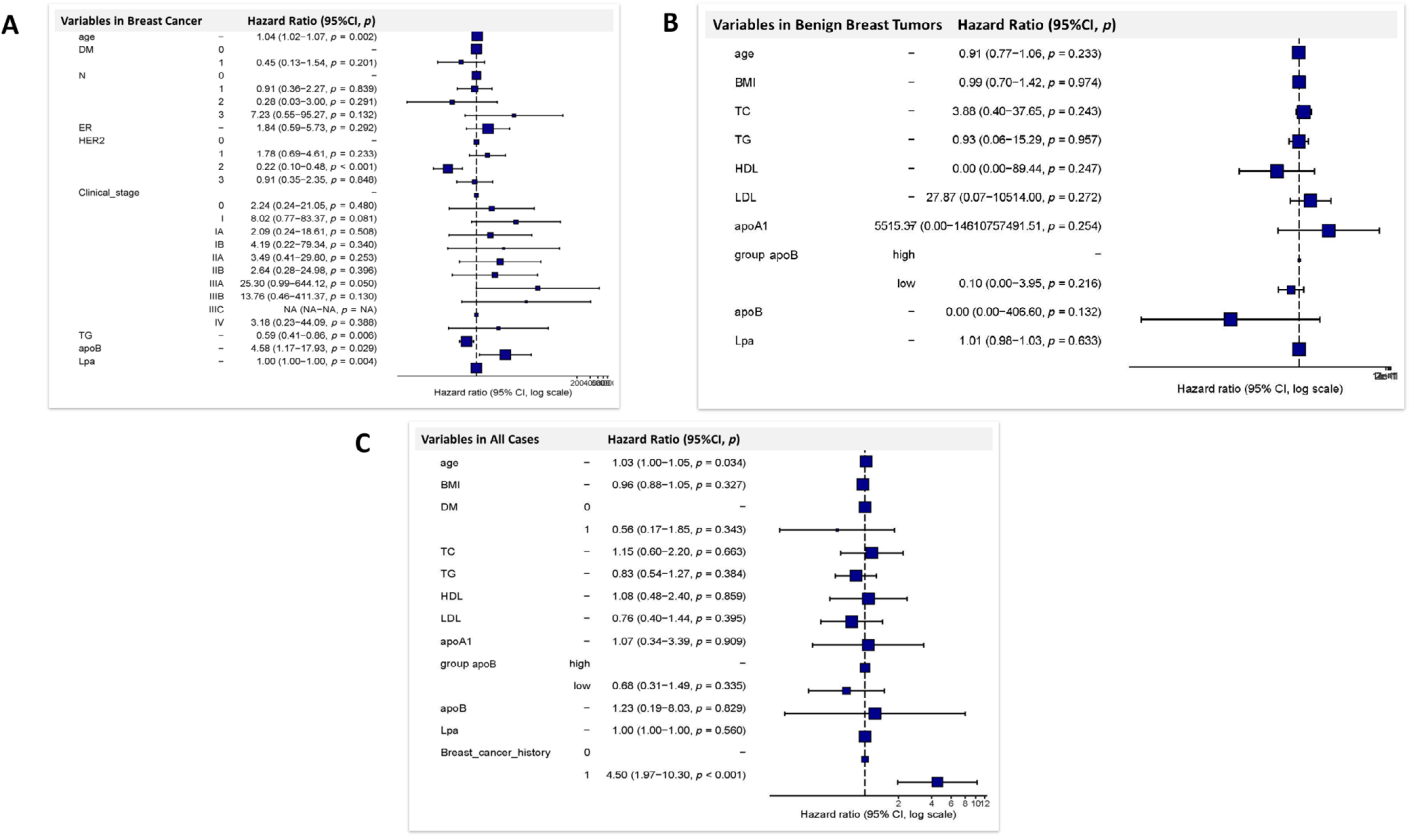
Forest plots for the multivariable Cox regression analysis of the factors affecting myocardial ischemia occurrence in breast cancer patients (A.), benign breast tumor patients (B) and the pooled cases (C).

In order to screen the factors responsible for myocardial ischemia occurrence, firstly, the univariate and multivariable Cox regression screening was performed in the factors for the breast cancer patients. The univariate regression suggested that age, TNM stages, clinical stages, and pathological categories were potential factors (*p* < 0.10). Regarding the clinical importance of cardiovascular events occurrence, the lipid indicators, and lipid‐lowering medications were also enrolled in the multivariable Cox regression analysis (Table 3). Results demonstrated that age, HER2, TG, ApoB, and Lp(a) were statistically significant factors in affecting myocardial ischemia occurrence. Considering the hazard ratio (HR), we finally included age (HR 1.04, 95% CI 1.02–1.07, *p* = 0.002), HER2 (HR 0.22, 95% CI 0.10–0.48, *p* < 0.001), TG (HR 0.59, 95% CI 0.41–0.86, *p* = 0.006) and ApoB (HR 4.58, 95% CI 1.17–17.93, *p* = 0.029) in the following analysis for myocardial ischemia in breast cancer patients (Table 3 and Figure 2A).

**TABLE 3:** The univariate and multivariable cox regression to screen the factors for myocardial ischemia in breast cancer patients.

Variables		HR (univariable)		HR (multivariable)	
		HR (95% CI)	*p*	HR (95% CI)	*p*
Demographics					
age		1.02 (1.00–1.04)	0.088	1.04 (1.02–1.07)	0.002*
weight		0.99 (0.96–1.02)	0.606		
height		1.00 (0.96–1.04)	0.864		
BMI		0.97 (0.90–1.06)	0.526		
Comorbidities					
anemia		0.84 (0.26–2.67)	0.765		
thyroid dysfunction		1.00 (0.60–1.68)	0.989		
hypertension		0.70 (0.32–1.52)	0.366		
hyperlipidemia		0.87 (0.46–1.67)	0.681		
DM		0.54 (0.17–1.73)	0.304	0.45 (0.13–1.54)	0.201
TNM stages					
*T*	0	2.33 (0.30–18.43)	0.421		
	1	3.06 (0.42–22.50)	0.271		
	2	2.51 (0.34–18.74)	0.368		
	3	2.72 (0.30–24.32)	0.371		
	4	6.99 (0.73–67.45)	0.093		
	*x*	1.44 (0.09–22.98)	0.798		
*N*	1	0.91 (0.51–1.62)	0.750	0.91 (0.36–2.27)	0.839
	2	1.60 (0.72–3.58)	0.248	0.28 (0.03–3.00)	0.291
	3	0.87 (0.21–3.58)	0.842	7.23 (0.55–95.27)	0.132
*M*	0	2.70 (0.37–19.45)	0.325		
	1	4.19 (0.38–46.21)	0.243		
	*x*	3.78 (0.39–36.39)	0.249		
Clinical stages	0	3.80 (0.48–29.99)	0.206	2.24 (0.24–21.05)	0.480
	I	6.30 (0.77–51.22)	0.085	8.02 (0.77–83.37)	0.081
	IA	4.64 (0.60–35.71)	0.140	2.09 (0.24–18.61)	0.508
	IB	4.25 (0.27–68.02)	0.306	4.19 (0.22–79.34)	0.340
	IIA	4.67 (0.62–35.21)	0.135	3.49 (0.41–29.80)	0.253
	IIB	2.98 (0.37–23.79)	0.304	2.64 (0.28–24.98)	0.396
	IIIA	8.33 (1.02–67.81)	0.048	25.30 (0.99–644.12)	0.050
	IIIB	6.00 (0.37–95.94)	0.205	13.76 (0.46–411.37)	0.130
	IIIC	3.86 (0.35–42.54)	0.271	—	—
	IV	8.93 (0.93–86.08)	0.058	3.18 (0.23–44.09)	0.388
Pathology					
ER+		2.11 (0.73–6.15)	0.169	1.84 (0.59–5.73)	0.292
PR+		2.19 (0.73–6.58)	0.164		
HER2	1+	2.22 (0.93–5.29)	0.071	1.78 (0.69–4.61)	0.233
	2+	0.34 (0.17–0.67)	0.002	0.22 (0.10–0.48)	0.001*
	3+	0.94 (0.40–2.20)	0.878	0.91 (0.35–2.35)	0.848
Ki67+		0.95 (0.79–1.14)	0.577		
Lipid					
TC		1.13 (0.90–1.42)	0.305	2.69 (0.90–8.00)	0.075
TG		0.99 (0.76–1.30)	0.953	0.59 (0.41–0.86)	0.006*
HDL		1.15 (0.66–1.99)	0.618		
LDL		1.09 (0.82–1.44)	0.566		
apoA1		1.25 (0.52–3.00)	0.614		
apoB		2.02 (0.70–5.79)	0.192	4.58 (1.17–17.93)	0.029*
Lpa		1.00 (1.00–1.00)	0.249	1.00 (1.00–1.00)	0.004*
Lipid‐lowering medication		1.26 (0.76–2.10)	0.377		
Atorvastatin		1.57 (0.85–2.87)	0.146		
Rosuvastatin		0.85 (0.31–2.35)	0.759		
Simvastatin		0.37 (0.09–1.50)	0.163		
Fluvastatin		2.87 (0.70–11.79)	0.144		
Pivastatin		0.53 (0.07–3.80)	0.526		
Ezetimibe		0.00 (0.00‐Inf)	0.997		
PCM		2.68 (0.84–8.56)	0.096		

Abbreviations: BMI, body mass index; DFS, disease‐free survival; DM, diabetes mellitus; ER, estrogen; HDL, high‐density lipoprotein cholesterol; LDL, low‐density lipoprotein cholesterol; PCM, patent Chinese medicine; PR, progesterone; TC, total cholesterol; TG, triglyceride.

* *p* < 0.05.

Next, for the factors associated with benign breast tumor patients, both univariate and multivariable Cox regression were conducted. Results of the univariate and multivariable Cox regression excluded all the factors with the results of *p* > 0.05. The ApoB also showed a *p* value of 0.132 in the multivariable Cox regression (Table 4 and Figure 2B). Furthermore, the factors in the pooled cases including both the breast cancer patients and benign breast tumor patients were screened. Results from univariate Cox regression showed that age, TC, and breast cancer history were statistically significant factors in affecting myocardial ischemia occurrence in the pooled patients. The multivariable Cox regression and forest plot indicated age (HR 1.03, 95% CI 1.00–1.05, *p* = 0.034) and breast cancer history (HR 4.50, 95% CI 1.97–10.30, *p* < 0.001) were significant affecting factors for the myocardial ischemia occurrence in the pooled patients (Table 5 and Figure 2C).

**TABLE 4:** The univariate and multivariable cox regression to screen the factors for myocardial ischemia in benign breast tumor patients.

Variables	HR (univariable)		HR (multivariable)	
	HR (95% CI)	*p*	HR (95% CI)	*p*
Demographics				
age	0.97 (0.89–1.05)	0.448	0.91 (0.77–1.06)	0.233
BMI	0.85 (0.61–1.17)	0.321		
Lipid				
TC	1.12 (0.46–2.69)	0.807	3.88 (0.40–37.65)	0.243
TG	0.75 (0.13–4.39)	0.751	0.93 (0.06–15.29)	0.957
HDL	1.56 (0.11–22.36)	0.743		
LDL	0.85 (0.35–2.04)	0.717		
apoA1	1.93 (0.05–78.60)	0.729		
apoB	0.38 (0.02–7.83)	0.532	0.00 (0.00–406.60)	0.132
Lpa	0.99 (0.98–1.01)	0.557	1.01 (0.98–1.03)	0.633

Abbreviations: BMI, body mass index; HDL, high‐density lipoprotein cholesterol; LDL, low‐density lipoprotein cholesterol; TC, total cholesterol; TG, triglyceride.

**TABLE 5:** The univariate and multivariable Cox regression to screen the factors for myocardial ischemia in the pooled breast cancer and benign breast tumor patients.

Variables		HR (univariable)		HR (multivariable)	
		HR (95% CI)	*p*	HR (95% CI)	*p*
Demographics					
age	1.04 (1.02–1.06)		< 0.001	1.03 (1.00–1.05)	0.034
BMI	0.97 (0.89–1.05)		0.483		
Lipid					
TC	1.26 (1.00–1.58)		0.047	1.15 (0.60–2.20)	0.663
TG	1.12 (0.88–1.43)		0.354	0.83 (0.54–1.27)	0.384
HDL	1.31 (0.73–2.35)		0.361		
LDL	1.15 (0.87–1.51)		0.320		
apoA1	1.80 (0.74–4.39)		0.193		
apoB	1.91 (0.76–4.78)		0.167	1.23 (0.19–8.03)	0.829
Lpa	1.00 (1.00–1.00)		0.490	1.00 (1.00–1.00)	0.560
Breast cancer history	5.36 (2.45–11.72)		< 0.001	4.50 (1.97–10.30)	< 0.001

Abbreviations: BMI, body mass index; HDL, high‐density lipoprotein cholesterol;LDL, low‐density lipoprotein cholesterol; TC, total cholesterol; TG, triglyceride.

Taking these results above, we compared the roles of ApoB in breast cancer patients, benign breast tumor patients, and pooled patients together, and the role of breast cancer history in affecting the myocardial ischemia occurrence. It was observed that ApoB was more important in breast cancer patients, rather than in benign breast tumor patients, to predict the myocardial ischemia occurrence (Tables 3, 4, 5 and Figure 2).

### 3.3 Construction of the Clinical Prediction Model Using the Screened Factors in Cox Regression

**FIGURE 3:**
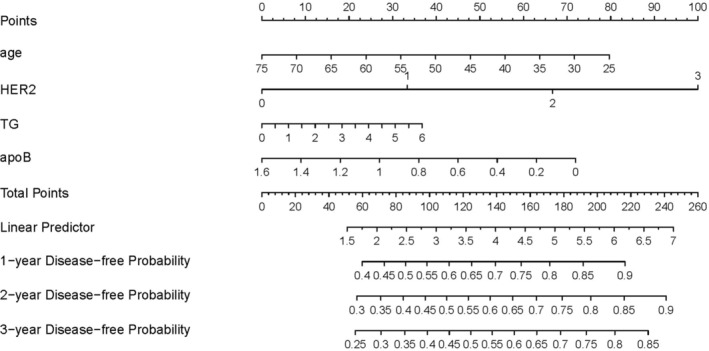
Nomogram plot for the selected factors affecting myocardial ischemia occurrence in breast cancer patients based on multivariable Cox regression analysis.

**FIGURE 4:**
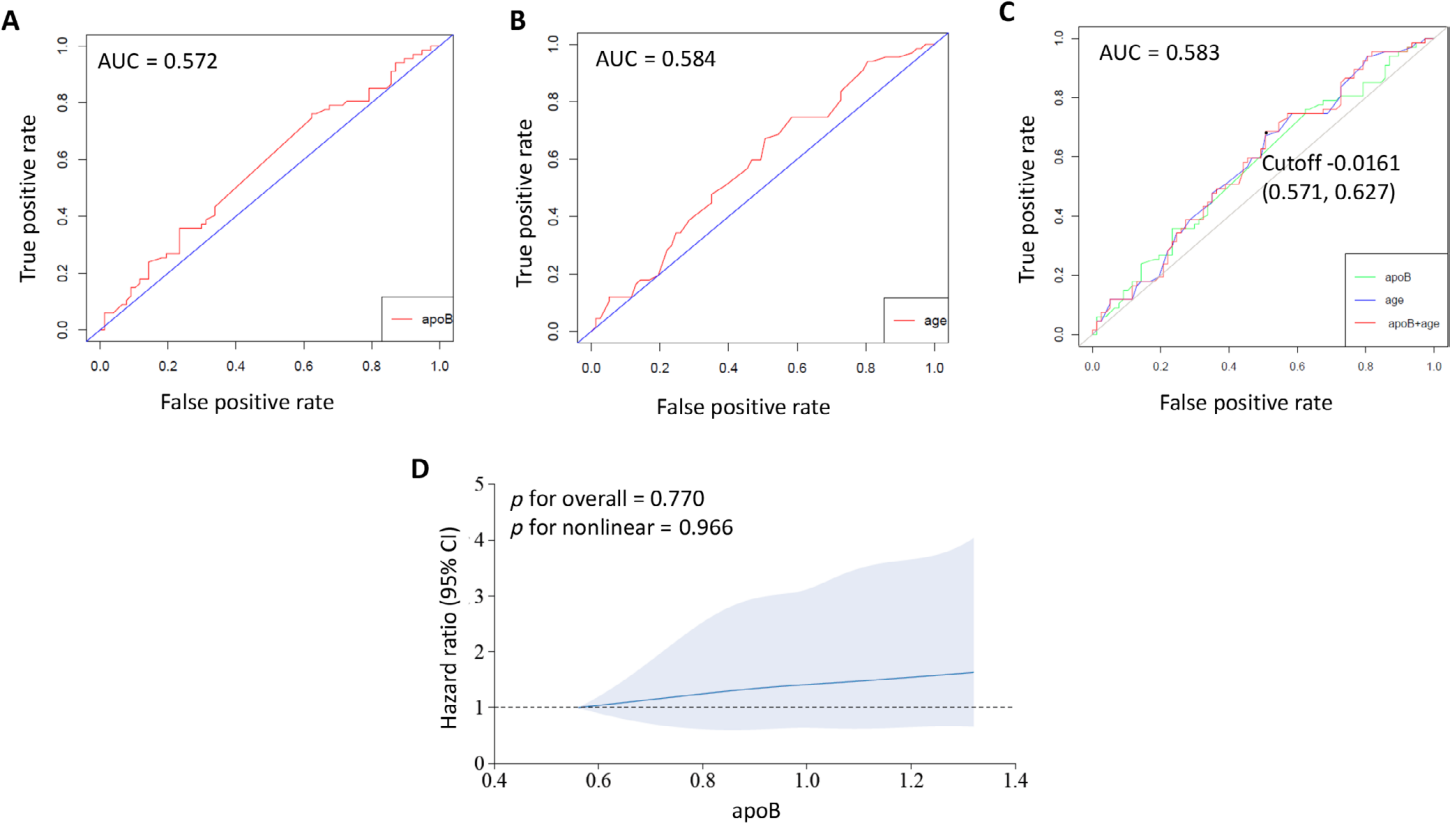
The receiver operating characteristic (ROC) curve plot and restricted cubic spline (RCS) plot of the selected factors affecting myocardial ischemia occurrence in breast cancer patients. ROC plot with area under curve (AUC) indicated of age (A), ApoB (B) and the combination of age and ApoB (C) in the prediction (D). The RCS plot for the association of ApoB with myocardial ischemia occurrence with 95%CI indicated by background color beside the curve.

The nomogram of the clinical prediction model was then constructed based on the selected factors in the multivariable regression, and the disease‐free probability within 3 years was predicted by measuring the points in each factor (Figure 3). The key factors involved in this prediction model were assessed by the ROC analysis and AUC calculation. It is observed that the AUC of ApoB and age was 0.572 and 0.584, respectively. The combination AUC of ApoB and age was 0.583 (Figure 4A–C). Then the RCS analysis was used to evaluate the nonlinear relationship in this model, and the result suggested that there was a linear association between ApoB and the HR of myocardial ischemia occurrence, and the HR increased in the interval of ApoB value between 0.6 an 1.3 (Figure 4D).

**FIGURE 5:**
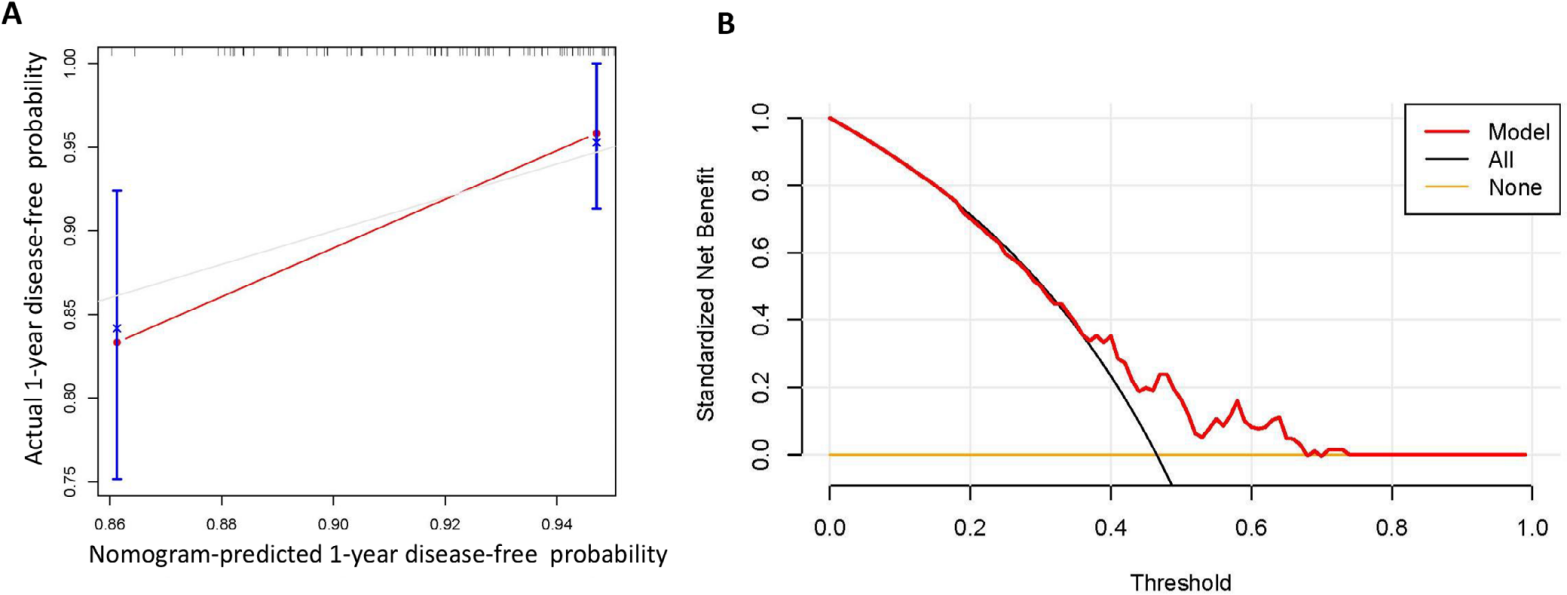
The calibration curve (A) and the decision curve analysis (DCA) (B) plots for the selected factors affecting myocardial ischemia occurrence in breast cancer patients based on multivariable Cox regression analysis.

In order to validate the predicted effect of the constructed model, the bootstrap algorithm was used for performing internal validation. The calibration curve showed that the nomogram model‐predicted 1‐year disease‐free probability was almost consistent with the actual probability line (Figure 5A). Then the DCA was performed to investigate the clinical net benefit in this prediction model, and the result suggested that there would be therapeutic clinical net benefit in the model‐predicted population than treating all the population or no treating for all the population in the predicted threshold interval was 0.35–0.70 (Figure 5B).

### 3.4 Clinical Validation of the Factors in the Prediction Model

**FIGURE 6:**
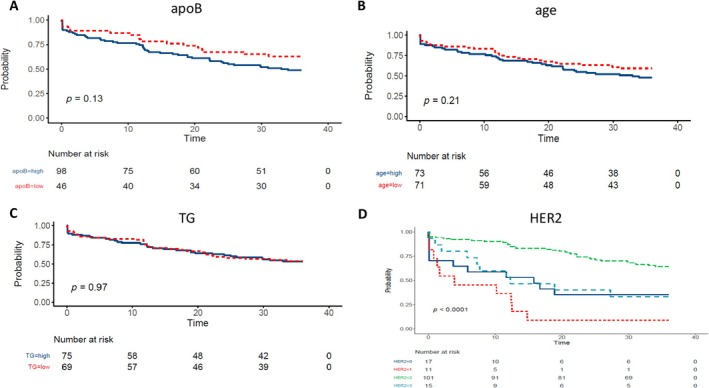
The Kaplan–Meier (KM) plot for cumulative occurrence probability in different categories of select factors, which were indicated to affect myocardial ischemia occurrence in breast cancer patients based on multivariable Cox regression analysis.

To further validate the clinical prediction value of factors screened for the prediction model, we performed the KM analysis based on the factors involved in the multivariable regression and the clinical outcomes. The result showed that during the 3‐year follow‐up, different HER2 subgroups led to a significantly different probability of myocardial ischemia occurrence, and the HER2^+^ category led to the most myocardial ischemia occurrence (*p* < 0.05). Although the *p* value in ApoB subgroups did not reach statistical significance, it is observed that the ApoB^high^ category lead to more disease incidents for myocardial ischemia (*p* = 0.13). A similar result was found in age subgroups (*p* = 0.21) (Figure 6). For the 3‐year DFS, there was no significant difference or even a trend of difference between the ApoB^low^ and ApoB^high^ subgroups (*p* = 1.000) (Table 1). These data indicated that the factors involved in the established model like HER2 and ApoB were helpful for the myocardial ischemia occurrence prediction in breast cancer patients.

## 4 Discussion

This clinical study was designed to investigate the factors affecting myocardial ischemia risk in breast cancer patients by developing a clinical predictive model and utilizing univariate and multivariate Cox regression analysis. We found that ApoB, age, and HER2 were the key factors responsible for disease incidence, which were identified as risk factors associated with the development of CVD in breast cancer patients. By comparing the significance of ApoB in breast cancer patients, benign breast tumor patients, and the combined patient group [15], as well as assessing the impact of the history of breast cancer on myocardial ischemia occurrence, we observed that ApoB was a more crucial predictor of myocardial ischemia in breast cancer patients compared to those with benign breast tumors. These factors above were used to construct the clinical prediction model based on the multivariable regression, and the KM plot indicated HER2^+^ category and ApoB^high^ were high‐risk populations for myocardial ischemia in breast cancer patients. However, there was no significant difference between the ApoB^low^ and ApoB^high^ subgroups in the 3‐year DFS.

It was reported that the lipid profile in breast cancer patients was different from that in benign breast tumors [15]. Regarding our finding that ApoB was important for the prediction of myocardial ischemic risk in breast cancer patients, the role of ApoB in CVDs and cancers was explored at first. ApoB serves as a key constituent of LDL‐C and plays a critical role in lipid metabolism and transportation [16]. Previous studies have established a link between elevated ApoB levels and a heightened risk of coronary heart disease and other ischemic CVDs [9, 11]. The ratio of ApoB to apoA1 (ApoB/apoA1) is frequently used as a marker to assess the risk of coronary heart disease associated with lipoproteins [17, 18]. Elevated levels of lipid ApoB have been implicated in atherosclerosis formation, thereby increasing the risk of CVD [9], and these reports were consistent with the results of our study. Moreover, ApoB plays a pivotal role in cholesterol transportation to peripheral tissues and is associated with breast cancer development [19]. Notably, the different roles of ApoB in the risk prediction of cardiovascular comorbidities of breast cancer and benign breast tumor patients were uncovered in this study.

Breast cancer is the most common malignancy affecting females, while CVD is known to have the highest rate of mortality. Several risk factors affecting both CVD and breast cancer are revealed, such as diet, smoking, and obesity [20]. It is reported that patients with early‐stage breast cancer exhibit an augmented susceptibility to CVD and mortality [21]. Certain studies have suggested a potential association between specific breast cancer pathological subtypes, such as human HER2^+^, and an elevated risk of CVD [22]. HER2 is a protein that is frequently overexpressed in certain breast cancers, which contributes to tumor development and progression within specific breast cancer subgroups [23]. Research has indicated that patients with HER2^+^ breast cancer exhibit an elevated risk of CVD, including coronary heart disease [20]. The risk may be influenced by the level of HER2 expression, with higher expression levels correlating with an increased risk of CVD [24].

Additionally, advanced age is a well‐established risk factor for CVD. Breast cancer patients, particularly the elderly, may face an even greater risk of CVDs [25]. Age‐related physiological changes and the presence of comorbidities may contribute to the increased risk of CVDs [26] in breast cancer patients [27]. Moreover, several studies have reported an association between elevated lipid levels (e.g., LDL, ApoB, Lpa) and an increased risk of breast cancer in patients [12, 28, 29]. Elevated lipid levels could potentially contribute to disease progression, inflammation, and adverse outcomes in breast cancer patients [30]. However, further research is necessary to establish a direct causal relationship between lipid levels and mortality within this specific population.

Above all, regarding the knowledge gap of whether myocardial ischemia risk factors differ in breast cancer and benign breast tumor patients [31], this clinical study tried to investigate the factors affecting myocardial ischemia risk in breast cancer patients. Our data demonstrated that ApoB and HER2 were potential factors in predicting the myocardial ischemia occurrence in breast cancer patients, rather than in benign breast tumor patients. This study will help screen high‐risk patients with breast cancer for the comorbidity occurrence of myocardial ischemia diseases by evaluating ApoB and HER2 levels, together with the routine indicators.

### 4.1 Limitations and Prospects

While this study provides insights into the relationship between serum ApoB and HER2 levels and myocardial ischemia risk in breast cancer patients, it is important to acknowledge its limitations. Firstly, the main limitation is the relatively small number of included cases (194 cases). This may have an impact on the statistical power and generalizability of the findings. A larger sample size would provide more robust results and enhance the reliability of the study [32]. Secondly, the limitation is the relatively short follow‐up period of 3 years. Myocardial ischemia risk in breast cancer patients may evolve over a longer time frame, and a longer follow‐up duration would allow for a more comprehensive evaluation of the relationship between serum ApoB and HER2 levels and myocardial ischemia risk [33]. Thirdly, benign tumors were used as a pseudo baseline for comparison with the breast cancer in this study. The reason for choosing benign tumors as controls for malignant tumors was that benign tumor patients had physiological and pathological foundations similar to healthy populations, but did not involve the complex biological processes and clinical manifestations unique to malignant tumors. Although benign breast tumors were presented as the control group in this study, a true control as close to normal tissue morphology and physiology should be chosen in further studies. Finally, the retrospective nature of this study introduces inherent biases and limitations. The reliance on medical records and data collected retrospectively may result in incomplete or inaccurate information. Additionally, the lack of control over the exposure and potential confounding variables may affect the internal validity of the study.

In this way, future research should focus on conducting a prospective randomized controlled trial (RCT) with a larger sample size, longer follow‐up duration, and diverse populations to validate these findings. RCT provides a higher level of evidence and allows for better control of confounding factors. Moreover, the current study focused on breast cancer patients in southern China, and it would be beneficial to include a more diverse population to assess the generalizability of the results. Including patients from different ethnic backgrounds and with varying comorbidities would provide a more comprehensive understanding of this result. Further research studies should elucidate the underlying mechanisms linking serum ApoB and HER2 levels and myocardial ischemia risk in breast cancer patients, including investigating the molecular pathways associated with this relationship.

## 5 Conclusion

This study constructed a clinical prediction model using three key factors (ApoB, age, and HER2) for myocardial ischemia incidence based on the multivariable regression analysis. This study demonstrated that ApoB and HER2 were potential factors in predicting the myocardial ischemia occurrence in breast cancer patients, rather than in benign breast tumor patients.

## Author Contributions

**Yeyan Lei:** data curation (lead), resources (lead), writing – original draft (supporting), writing – review and editing (lead). **Dongmei Li:** data curation (equal), resources (equal). **Shuang Bai:** data curation (equal), resources (equal). **Xing Zeng:** data curation (equal), formal analysis (equal), visualization (equal), writing – original draft (equal). **Rongyuan Yang:** supervision (equal), validation (equal). **Qing Liu:** conceptualization (equal), funding acquisition (equal), methodology (equal), project administration (equal), resources (equal), supervision (equal), validation (equal), writing – original draft (equal), writing – review and editing (equal).

## Ethics Statement

Approval of the research protocol by an Institutional Reviewer Board: This study was approved by the Ethics Committee of Guangdong Provincial Hospital of Traditional Chinese Medicine. Informed Consent: N/A. Registry and the Registration No. of the study/trial: No. ZE2023‐318. Animal Studies: N/A.

## Conflicts of Interest

The authors declare no conflicts of interest.

## Supporting information


Figure S1.

## Data Availability

The authors have nothing to report.
