# Supplementary material for: Clinical Relationship Between Serum ApoB, HER2, and Myocardial Ischemia Risk in Breast Cancer Patients
Source: Cancer Rep (Hoboken). 2025 Jul 18;8(7):e70075. doi: 10.1002/cnr2.70075 (PMC12272302; doi:10.1002/cnr2.70075)
Supplement: Supplementary file 1 — Figure S1. [file CNR2-8-e70075-s001.pdf]

- **Figure S1 legend**
- The Kaplan-meier (KM) plot for cumulative occurrence probability in different categories of select factors, which were indicated to affect myocardial ischemia occurrence in breast cancer patients based on multivariable cox regression analysis.

Figure S1

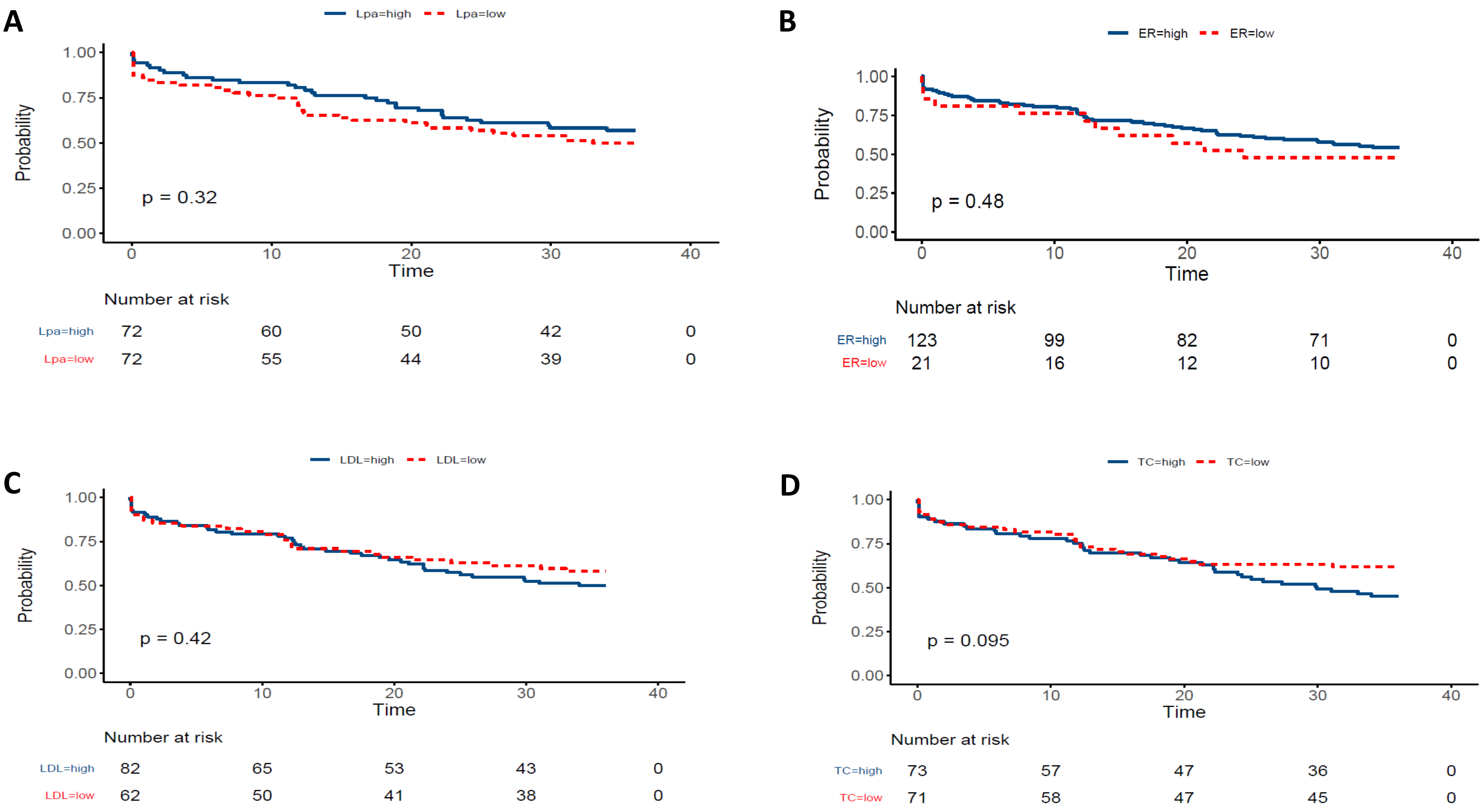
A
Lpa=high
Lpa=low
Probability
1.00
0.75
0.50
0.25
0.00
p = 0.32
0
10
20
30
40
Time
Number at risk
Lpa=high 72 60 50 42 0
Lpa=low 72 55 44 39 0
B
ER=high
ER=low
Probability
1.00
0.75
0.50
0.25
0.00
p = 0.48
0
10
20
30
40
Time
Number at risk
ER=high 123 99 82 71 0
ER=low 21 16 12 10 0
C
LDL=high
LDL=low
Probability
1.00
0.75
0.50
0.25
0.00
p = 0.42
0
10
20
30
40
Time
Number at risk
LDL=high 82 65 53 43 0
LDL=low 62 50 41 38 0
D
TC=high
TC=low
Probability
1.00
0.75
0.50
0.25
0.00
p = 0.095
0
10
20
30
40
Time
Number at risk
TC=high 73 57 47 36 0
TC=low 71 58 47 45 0
